# Dental Education

**Published:** 1884-06

**Authors:** 


DENTAL EDUCATION.—The subject of dental education has engaged the attention of the profession from the time dentistry emerged from the condition of a mere trade to its present status.

Almost every number of the various Dental Journals, and the proceedings of every Dental Society, present essays and editorials, giving the individual views of the writers, many of whom suppose that the plan they promulgate is the only feasible one, while others declaim against any system which is not propagated by themselves, or in the carrying out of which they have not a part. Another class of writers or speakers, condemn all methods of collegiate instruction, simply because they have not enjoyed such privileges themselves, being content to pursue the beaten path, old and absolute though it may be, which they have followed for many years, without an effort to improve with the profession they practice.

Although great diversity of opinion may exist even among those qualified to judge, as to the manner dental schools should be conducted, yet a large majority of the most eminent dental practitioners recognize the fact that the *reputable* dental schools at least, are doing all in their power, and are accomplishing a noble work, equal in all respect to that emanating from the schools of the kindred professions. That abuses have been perpetrated by certain schools, some of them schools whose previous standing should be a guananted against any retrogression, is beyond question, and this fact has caused certain Journals, independent and outspoken, to publicity notice what has for a long time been a matter of common report. The passage of State laws regulating the practice of dentistry, and the appointment of State examining boards to carry out the provisions of such laws, have been instrumental in bringing about a surveilance, which must be beneficial, and act as a check against the indiscriminate issuing of diplomas, withou regard to time of study and qualification.

The Buchanan system of selling diplomas broadcast, may not now be pursued with the unblushing effrontery which characterized that fradulent scheme, except in the well-known Wisconsin case, but a practice which has very little more to commend it, has been pursued by some schools of longer existence, and which has led to the present agitation.

The best interests of the profession demand that every dental school should not only publish its requirements for graduation, but should strictly adhere to such requirements.

If the revenue derived from an upright, honorable course, such as should be pursued in conducting every reputable dental school, is not sufficient for its support, then it would be greatly to the honor of all concerned that such a school should cease to exist, rather than attempt to prolong its life by graduating students irregularly.

We trust that the good effects even now apparent in the administration if the State laws regulating the practice of dentistry, may not only continue, but increase, and that the State Boards of dental examiners may, by the exercise of the powers conferred upon them exercise great good, and correct many irregularities which may now exist in the method of Collegiate instruction.

